# Cancer vaccines: comprehensive review

**DOI:** 10.1093/bjsopen/zrag041

**Published:** 2026-05-29

**Authors:** Rachael E Clifford, Nichola Manu, Qian Yang, Zeeshan Arif, David Aggen, Michael E Bryan, Aditi Gupta, Heather Shaw, David Church, Philippa Corrie, Natalia Savelyeva, Christian Ottensmeier, Sarah Danson, Robert Jones

**Affiliations:** Colorectal and Peritoneal Oncology Centre (CPOC), The Christie NHS Foundation Trust, Manchester, UK; The Liverpool Head and Neck Centre, The University of Liverpool, Liverpool, UK; Centre for Human Genetics, Nuffield Department of Medicine, University of Oxford, Oxford, UK; Colorectal and Peritoneal Oncology Centre (CPOC), The Christie NHS Foundation Trust, Manchester, UK; Genitourinary Oncology Service, Department of Medicine, Memorial Sloan Kettering Cancer Centre, New York, New York, USA; Centre for Immuno-Oncology, University of Oxford, Oxford, UK; Cancer Program, Broad Institute of MIT and Harvard, Cambridge, Massachusetts, USA; Genitourinary Oncology Service, Department of Medicine, Memorial Sloan Kettering Cancer Centre, New York, New York, USA; Department of Medical Oncology, University College London Hospital, London, UK; Department of Medical Oncology, Mount Vernon Cancer Centre, London, UK; Centre for Immuno-Oncology, Nuffield Department of Medicine, University of Oxford, Oxford, UK; Cambridge University Hospitals NHS Foundation Trust, Cambridge, UK; The Liverpool Head and Neck Centre, The University of Liverpool, Liverpool, UK; The Liverpool Head and Neck Centre, The University of Liverpool, Liverpool, UK; Division of Clinical Medicine, School of Medicine and Population Health, University of Sheffield, Sheffield, UK; UK Vaccine Innovation Pathway, National Institute for Health and Care Research, London, UK; The Liverpool Head and Neck Centre, The University of Liverpool, Liverpool, UK

## Abstract

**Background:**

There has been an increasing focus on the potential of cancer vaccines as a therapeutic oncological option, aiming to harness the immune system to recognize and eliminate malignant cells. Unlike traditional therapies, vaccines exploit tumour-associated antigens and neoantigens to generate durable, tumour-specific immune responses.

**Methods:**

This review provides a comprehensive overview of current vaccine strategies, their clinical applications, and implications for surgical oncology.

**Results:**

Clinical trials highlight promising results in high-mutational burden cancers such as melanoma, lung, and bladder cancer, as well as in traditionally ‘cold’ tumours like pancreatic cancer. Combination strategies, particularly with checkpoint inhibitors, have shown enhanced efficacy, exemplified by messenger RNA-4157/V940 and autogene cevumeran in adjuvant settings. Off-the-shelf tumour-associated antigen vaccines offer scalability but face challenges of tolerance and limited specificity. Early data suggest vaccines may improve relapse-free survival and induce antigen spreading, broadening immune responses beyond the initial targets.

**Conclusion:**

Cancer vaccines are poised to complement surgery and systemic therapies, particularly in the perioperative window. Their integration into multimodal treatment strategies may redefine precision oncology, offering durable disease control and improved patient outcomes.

## Teaser

There has been an increasing focus on the potential of cancer vaccines as a therapeutic oncological option, aiming to harness the immune system to recognize and eliminate malignant cells. This review provides a comprehensive overview of current vaccine strategies, their clinical applications, and implications for surgical oncology.

## Introduction

Cancer vaccines for solid tumours have gained increasing attention over recent years. Tumour growth is driven by mutations that promote uncontrolled cell survival and proliferation, and understanding these mutations has been essential for developing targeted treatments and personalized medicine^1^. Some of these mutations produce abnormally expressed surface proteins that the immune system can recognize as foreign, paving the way for novel treatments that harness the inherent weaknesses in cancer immunology.

Cancer immunology focuses on how the immune system detects and controls tumour cells. The immune system consists of innate and adaptive branches. Whereas the innate immune system provides an immediate response, the adaptive immune system, particularly T cells, plays a key role in long-term recognition and destruction of tumour cells. Tumour-associated antigens (TAAs) are proteins expressed at higher levels in cancer cells than normal tissues, whilst neoantigens are unique to tumour cells due to DNA mutations. These antigens can be presented to T cells by antigen-presenting cells, particularly dendritic cells, which initiate an immune response. However, many tumours evade immune detection by suppressing immune checkpoints, altering antigen presentation, or creating an immunosuppressive microenvironment. Immunotherapies, including vaccines, aim to overcome these barriers by enhancing antigen presentation and T cell activation. Achieving this requires effective delivery of tumour-specific proteins (antigens) to dendritic cells, stimulation of CD4^+^ helper T cells and cytotoxic T lymphocytes (CTLs), infiltration of the tumour environment, and sustained immune response^2^.

Prophylactic cancer vaccines targeting oncogenic viruses represent the most clinically successful vaccine strategy to date. Human papillomavirus (HPV) vaccines have led to marked reductions in cervical cancers, and the hepatitis B virus (HBV) vaccination has led to substantial population-level declines in hepatocellular carcinoma incidence. The hepatitis C virus (HCV), although lacking a licensed vaccine, illustrates the preventative paradigm whereby viral eradication through antivirals significantly reduces downstream cancer risk, reinforcing the principle that immune targeting of oncogenic drivers can prevent malignancy.

This review article aims to serve as a primer for surgeons and clinicians who are going to be treating patients managed with cancer vaccines to provide a comprehensive overview and framework for understanding these novel technologies.

## Types of cancer vaccines

Vaccines can be used in both prophylactic and therapeutic settings. Prophylactic vaccines aim to prevent cancer in patients who are currently disease-free, often by eliminating causative agents, whilst therapeutic vaccines are designed to enhance or reactivate the immune system in patients with existing disease. The first reported use of vaccines to treat cancer was by William Coley in the 1890s, who used streptococcal bacteria as a therapeutic vaccine for patients with sarcoma^3^. His approach was based on observations that certain infections appeared to reduce cancer incidence. Although interest in cancer vaccines waned with the rise of chemotherapy and radiotherapy, recent advances in tumour biology and the tumour microenvironment have led to renewed interest.

Cancer vaccines can be produced using a number of different approaches^4^. Whole-cell vaccines use tumour cells (autologous or allogeneic) that have been treated (usually irradiated) to prevent replication. They contain a broad range of antigens but often lack tumour specificity with marked self-overlap and are complex to produce. By contrast, peptide/protein-based vaccines use short or full-length tumour-specific peptides or proteins. They are relatively simple to produce and can target well defined antigens like New York esophageal squamous cell carcinoma-1 (NY-ESO-1) and melanoma-associated antigen 3 (MAGE-A3). However, they have limited immunogenicity, risk of immune tolerance as many target self-derived antigens, human leukocyte antigen (HLA) restriction reducing patient applicability, and poor induction of cytotoxic CD8^+^ T cell responses unless paired with strong adjuvants or delivery systems.

Patient-derived dendritic cells are loaded with tumour antigens and reinfused to initiate a strong immune response. Sipuleucel-T (Provenge®) is an approved example for prostate cancer. However, production is labour-intensive and costly, requiring *ex vivo* cell harvest, culture, and antigen loading under good manufacturing practice conditions. Variability in dendritic cell quality between patients affects reproducibility. Manufacturing is slow, limiting timely treatment. Clinical efficacy has generally been modest, and large-scale implementation is logistically challenging. Viral vector-based vaccines employ modified viruses (for example, adenovirus, poxvirus) to deliver tumour antigen genes, for example PROSTVAC in prostate cancer. However, pre-existing antiviral immunity may neutralize the vector, limiting efficacy; repeated dosing can be ineffective due to antivector responses; manufacturing is complex and costly; and safety concerns include systemic inflammation or off-target immune reactions. Clinical benefit has often been modest.

Nucleic acid vaccines (DNA/messenger (m)RNA)/mRNA vaccines gained prominence during the COVID-19 pandemic and offer a number of key advantages. These vaccines are highly adaptable and allow rapid production. They deliver the genetic code for tumour antigens (which can be patient-specific antigens or conserved antigens that are expressed in the majority of tumours of that type), which are then produced inside the body. By using low immunogenic vectors such as lipid envelopes, they are able to minimize immune elimination before the mRNA is synthesized within dendritic cells, which maximizes the T cell response. However, they face several disadvantages: some require ultra-cold storage and complex delivery systems; manufacturing costs remain high; immune responses can be variable, often short-lived without strong adjuvant treatments; there is potential for systemic reactogenicity; and large-scale personalized production poses enormous logistical challenges.

**Fig. 1:**
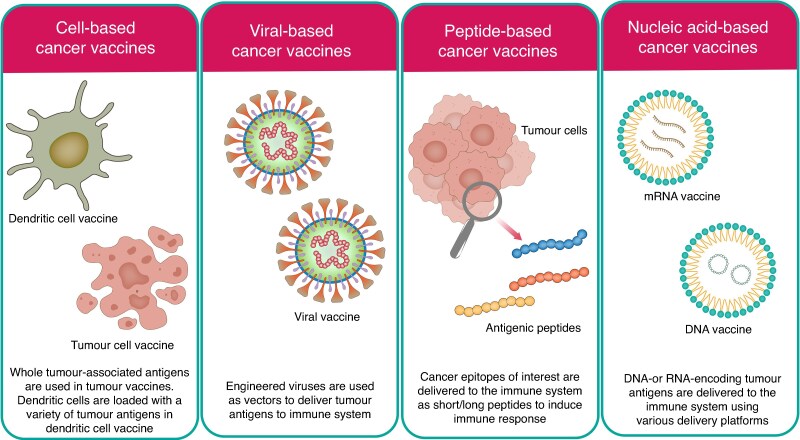
A figure taken from Vishweshwaraiah *et al.*^5^ demonstrating types of cancer vaccines and their mechanism of action

A summary of the different types of vaccines can be found in *Fig. 1*.

## Neoantigen selection for cancer vaccines

Neoantigens are small protein fragments that arise from tumour-specific mutations^6^. Unlike TAAs, which may also be found in normal tissues at low levels, neoantigens arise exclusively in cancer cells and are novel to the immune system^6,7^. This tumour specificity makes them ideal targets for personalized cancer vaccines: they are recognizable as ‘foreign’ without risking autoimmunity^8^. For surgeons, this presents a unique opportunity: the resected tumour itself becomes the raw material for highly tailored immunotherapy.

Identifying an effective neoantigen, however, remains challenging. Tumours may harbour hundreds of mutations, but only a fraction yield peptides that are expressed, processed, presented on major histocompatibility complex (MHC) molecules, and recognized by T cells; many mutations are immunologically silent. Some produce peptides that bind to MHC molecules but are unstable or ignored by the immune system. Others are obscured by tumour immune evasion. Including these ‘false positives’ can dilute vaccine efficacy, while excluding potent neoantigens may lead to missing the chance to trigger a robust response.

These challenges are particularly acute in the perioperative window. After resection, when disease burden is minimal and immune responsiveness may rebound following perioperative suppression, there is a brief but important period for potential intervention. Precision in neoantigen selection at this stage can directly influence vaccine success. The pipeline therefore begins during surgery with the collection of tumour and matched normal tissue for DNA and RNA sequencing. This allows for identification of tumour-specific somatic mutations and determination of which mutations are actually expressed at the RNA level. Bioinformatic tools can then efficiently predict which mutant peptides could be presented by the patient’s HLA molecules^9^. The result is a preliminary shortlist of candidate neoantigens. However, binding prediction alone does not guarantee a useful immune response; selecting the most promising neoantigen candidates requires a deeper understanding of how the immune system sees and responds to these peptides.

**Fig. 2:**
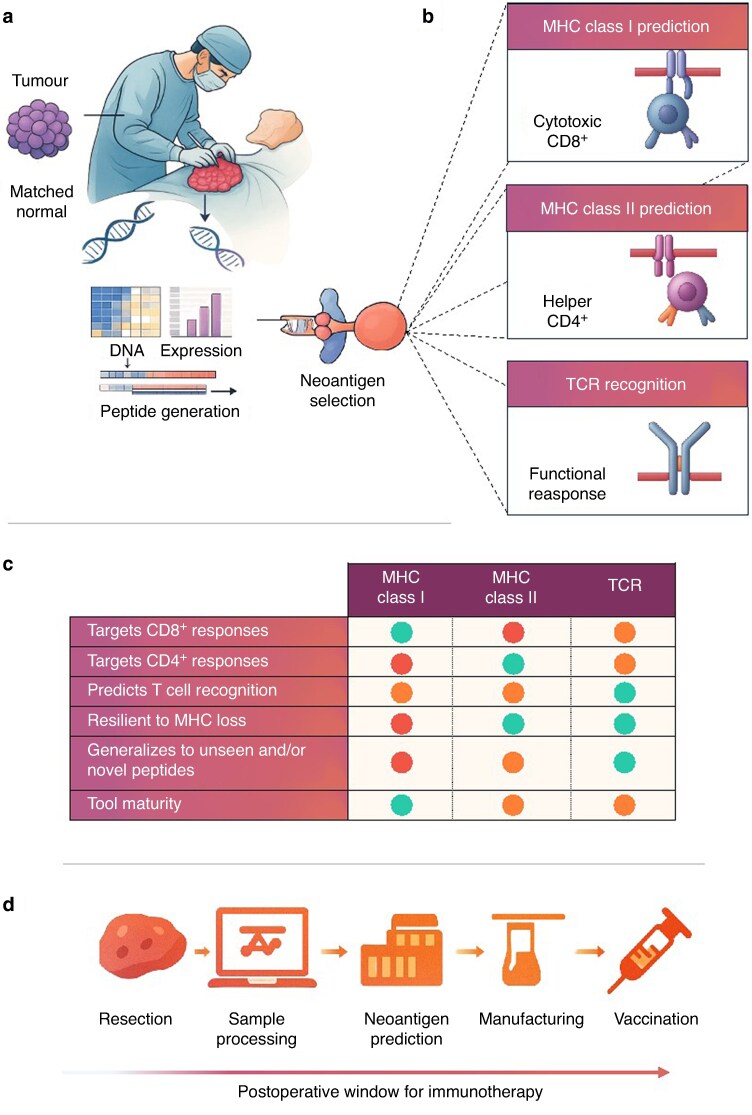
Neoantigen selection pathway and predictive approaches **a** Tumour and matched normal tissue are collected during resection for genomic and transcriptomic profiling. DNA and RNA sequencing enables identification of tumour-specific mutations and assessment of expression, forming the basis for neoantigen candidate generation. **b** Three major predictive strategies guide selection. Class I prediction identifies peptides likely to bind class I molecules and activate cytotoxic CD8^+^ T cells. Class II prediction targets longer peptides that engage CD4^+^ helper T cells and support broader immune responses. TCR recognition models focus on predicting whether presented peptides will be recognized by a TCR and elicit a functional immune response. **c** Comparison of predictive strategies across key features. Each method offers distinct strengths: class I and II predictions target different T cell subsets, whereas TCR-based models aim to capture downstream immunogenicity. Trade-offs include tool maturity, resilience to immune escape, and ability to generalize to novel peptides. **d** The neoantigen selection pipeline spans from surgical resection through sample processing, prediction, and vaccine manufacturing, all within a limited postoperative window for immunotherapy. Tissue quality and workflow efficiency during this period are important to vaccine success. MHC, major histocompatibility complex; TCR, T cell receptor.

Three predictive strategies have emerged to tackle this problem; the first focuses on MHC class I presentation, targeting peptides likely to activate cytotoxic CD8^+^ T cells^10^. The second includes MHC class II pathways to engage helper CD4^+^ T cells^11^. The third, more recent, models the interaction between the presented peptide and the T cell receptor (TCR). Each approach provides a unique perspective on neoantigen immunogenicity, with different assumptions, limitations, and implications for vaccine design. Understanding how and when to apply these approaches is key to translating tumour samples into effective, patient-specific therapies. A summary of the neoantigen selection pathway and predictive approaches is shown in *Fig. 2*.

### MHC class I selection

MHC class I prediction is the most established method for neoantigen selection and forms the foundation of most personalized cancer vaccine strategies. It focuses on identifying peptides presented on the surface of tumour cells via MHC class I molecules, where they may be recognized by CD8^+^ cytotoxic T cells. These T cells play a central role in antitumour immunity by directly destroying cancer cells that present abnormal peptides^12^.

Selection begins by identifying non-synonymous mutations, typically using whole-exome sequencing. These mutations are used to generate short peptide sequences, usually 8 to 11 amino acids in length, which are computationally assessed for their likelihood of binding to the patient’s specific MHC class I alleles. Tools such as NetMHCpan and MHCflurry estimate binding affinity, and peptides with high predicted binding are prioritized for further consideration^13–15^.

The main advantage of this approach is its specificity. Because class I molecules present peptides from intracellular proteins, the immune system can detect and eliminate cells that display tumour-specific mutations without affecting normal tissue. Moreover, CD8^+^ T cells are directly cytotoxic, making them highly effective at clearing cancer cells once activated. However, strong MHC binding alone does not always translate into immunogenicity. Many predicted peptides are never actually processed or presented, and others may be ignored by the immune system altogether^16^. Furthermore, some tumours downregulate MHC class I molecules or mutate key components of the antigen presentation pathway, making them invisible to CD8^+^ T cells^17^. These immune escape mechanisms can limit the effectiveness of class I-targeted vaccines.

Despite these challenges, this method remains a critical first step in neoantigen discovery. It is well supported by immunological data and has been used successfully in early clinical trials^18^. In high-mutational burden cancers such as melanoma, non-small cell lung cancer, and bladder cancer, class I-targeted vaccines offer a practical and personalized route to immunotherapy.

### MHC class II selection

Alongside class I strategies, there is growing interest in neoantigens presented by MHC class II molecules. These longer peptides (typically 1225 amino acids) are recognized by CD4^+^ helper T cells, immune cells that coordinate antitumour immunity, support cytotoxic CD8^+^ responses, and sustain immune memory^12^. Their role is especially important in tumours with impaired MHC class I presentation or immune evasion.

Class II molecules present peptides derived from extracellular or phagocytosed proteins (a potential route for ‘autovaccination’ by dying tumour cells). To identify targets, longer mutant sequences are generated and assessed for binding to patient-specific HLA class II alleles using tools such as NetMHCIIpan^11^. A feature of class II peptides is their promiscuity: the ability to bind to multiple HLA types, potentially enabling broader patient coverage and semi-personalized vaccine strategies.

Incorporating class II neoantigens can improve the quality and durability of CD8^+^ responses and stimulate the production of cytokines that shape the broader immune environment. In some trials, strong CD4^+^ responses were observed even when CD8^+^ responses were weak, highlighting their standalone therapeutic value^18^. This is particularly relevant for cancers like glioblastoma or pancreatic cancer, where class I presentation may be limited.

Prediction, however, is more complex. The flexible binding groove of MHC class II allows multiple overlapping registers, increasing uncertainty^19^. Additionally, class II expression is highly variable across tissues, and predictive models are less mature and supported by fewer training data than class I models, although improving.

For surgeons, these insights have practical implications. High-quality RNA, alongside DNA, is important for identifying viable class II candidates. The same tumour specimen collected for class I analysis can also yield class II targets, provided RNA integrity and sequencing depth are sufficient. Including both classes in vaccine design is believed to enhance the likelihood of a durable, multipronged immune response.

### TCR–epitope prediction

Although predicting MHC binding is an important step, it does not guarantee immune recognition. Many peptides predicted to bind MHC class I or II are never recognized by T cells. To bridge this gap, a new class of models shifts focus from presentation to recognition, aiming to predict whether a presented peptide will be seen by a TCR and elicit a functional response.

This reframes the question from ‘can it be displayed?’ to the more functionally relevant ‘will the immune system see it?’. Tools do this by comparing the features of the peptide to those of known TCRs that have been shown to recognize similar targets. Some tools, like DeepTCR and TCRMatch, rely on sequence similarities^20,21^, whereas models like TITAN apply deep learning to predict structural interactions between peptides and TCRs^22^.

Though still in development, these tools offer practical benefits. They narrow down the list of predicted neoantigens to those with the greatest chance of being seen by T cells. This is especially valuable in low-mutation tumours or when only a limited number of antigen slots are available, such as in mRNA- or peptide-based formulations. However, the data available to train these models are still sparse and biased towards common infections, rather than cancer-specific responses. Most tools are better at recognizing familiar patterns than predicting responses to novel ones^23^. Furthermore, they cannot yet fully capture immune complexity, including the influence of HLA type, TCR diversity, and tumour microenvironment.

Despite these limitations, as these models evolve they are likely to become a valuable addition to neoantigen selection pipelines. For the surgical team, their value lies in helping ensure that the peptides included in a patient’s vaccine are not just presentable, but likely to be seen and acted upon by the immune system^23^. In time-sensitive windows such as the perioperative period, this added layer of prioritization may be key to enhancing the impact of cancer vaccines.

Production timelines vary significantly dependent on platform type: off-the-shelf peptide can be produced within weeks, whereas personalized mRNA vaccines can require 6–12 weeks from tumour sampling to first-dose delivery because of demands for high-quality sequencing, epitope prediction, and manufacturing and quality control steps—another reason why the adjuvant timeline lends itself to these technologies.

Neoantigen prediction is increasingly refined. The precise number of high-quality HLA-restricted immunogenic targets identified varies widely by tumour mutational burden, and in practice a number of coexpressed antigens are required to avoid immune escape. Vaccines are produced in around 90% of patients in clinical vaccine trials, with attrition mainly due to insufficient genetic tissue extraction (highlighting the importance of perioperative tissue handling and processing).

## Cancer vaccines utilized as single therapy for solid organ malignancy

### Pancreatic cancer

Pancreatic cancers are typically characterized by a hostile immune microenvironment: they are generally devoid of immune effector cells, have a low tumour mutation burden, and express a paucity of quality neoantigens; in effect, they are ‘immune deserts’^24^. Cancer vaccines offer a strategy to enhance T cell activation and disrupt signals that downregulate T cell trafficking and function within the tumour microenvironment. Encouragingly, emerging data from early phase clinical trials suggest vaccines could meaningfully improve outcomes for patients with ‘cold tumours’ like pancreatic cancer over the coming decade. Based on the observation that long-term pancreatic cancer survivors appear to mount robust T cell responses against tumour-specific neoantigens that are not shared across patients^25^, Rojas *et al* used the BioNTech individualized RNA vaccine manufacturing platform to treat 16 patients with resected pancreatic cancer with the adjuvant ‘autogene cevumeran’ vaccine, generated from the tumour tissue removed at surgery. This vaccine was administered in combination with atezolizumab and mFOLFIRINOX (modified folinic acid, fluorouracil, irinotecan, and oxaliplatin) chemotherapy^26^. In this phase 1 trial, 50% of patients generated high levels of neoantigen-specific T cells, and these responders had significantly improved outcomes compared with non-responders. A recent study update at median follow-up of 3.2 years reported that the median relapse-free survival (RFS) in responders was still not reached, compared with the median RFS of 13.4 months in non-responders (hazard ratio (HR) 0.14; *P* = 0.007). Furthermore, vaccination appears to elicit durable T cell memory, with an average estimated lifespan of over 7 years for CD8^+^ T cells^27^. These promising findings are now being evaluated in a randomized phase II trial (NCT05968326) known as the IMcode003 trial, which is recruiting patients with resectable pancreatic adenocarcinoma before their surgery. Eligible patients are randomized to receive either standard adjuvant mFOLFIRINOX chemotherapy alone, or in combination with autogene cevumeran and atezolizumab. Global recruitment is slower than anticipated and reflects some of the challenges of developing personalized vaccines. They require involvement of multiple specialist teams, as well as patient willingness to enrol in research at a vulnerable time, facing the prospect of major surgery with uncertain outcomes with no guarantee of ultimately receiving the vaccine.

Alternatively, a more scalable approach may lie in targeting shared oncogenic mutations. Over 90% of pancreatic cancers harbour mutations in the *KRAS* gene, making it a compelling target for an ‘off-the-shelf’ vaccine, which would overcome some of the burdensome complexity and resource requirements associated with personalized vaccine manufacture. ELI-002 was developed as a lymph node-targeted peptide vaccine designed to train T cells to recognize and destroy cancer cells expressing *KRAS*-mutated proteins. The first-generation vaccine, ELI-002 2P, included mutant *KRAS* G12D and G12R peptides and was tested in the phase I AMPLIFY 201 trial^28^. In this study, 25 patients (20 with pancreatic cancer, 5 with colorectal cancer) exhibited minimal residual disease, defined as having radiologically occult disease but with either positive postoperative circulating tumour DNA (ctDNA) or raised tumour markers (CA19.9 or carcinoembryonic antigen), and received the vaccine. Seventeen patients had strong mutant *KRAS*-specific T cell responses (‘responders’), whereas eight had weaker responses (‘non-responders’). At median follow-up of 20 months, RFS was not reached in responders, but was 3 months in non-responders (*P* = 0.0002). Four of the 17 responders had died compared with 7 out of 8 non-responders. A randomized phase II trial (NCT05726864) testing a broader, seven peptide formulation of ELI-002 (ELI-002 7P: *KRAS*/*NRAS* G12D, R, V, S, A, C and G13D) in the adjuvant pancreatic cancer setting is currently underway.

Interestingly, after ELI-002 2P vaccine treatment, T cells reactive to personalized tumour antigens that were not present in the vaccine were identified in 67% of treated patients. This phenomenon known as ‘antigen spreading’ may contribute to the observed delay in tumour recurrence. This supports the rationale for exploring both shared-antigen and personalized vaccine approaches; however, their future application remains to be determined by the results of the ongoing randomized trials.

### Head and neck cancer

Head and neck squamous cell carcinoma (HNSCC) is the sixth most common cancer type worldwide with approximately 2.5 million cases and 379 000 deaths annually^29,30^. Approximately 50% of oropharyngeal squamous cell carcinomas (OPSCCs) with positive HPV are driven by the high-risk type HPV16^30^. These patients commonly present with locally advanced nodal disease yet have a 5-year overall survival (OS) of ∼ 70% compared with ∼ 40% in patients with HPV-negative HNSCC^31–34^. This survival benefit is attributed to a higher frequency of tumour-infiltrating lymphocytes, likely reflecting a partially protective adaptive anticancer immune response to HPV antigens. Nonetheless, at recurrence, or at presentation with metastatic disease, the survival benefit diminishes. Immunotherapy with antiprogrammed cell death protein 1 (aPD-1) antibodies enables long-term survival in about 15% of patients, with no clear advantage for HPV-positive *versus* HPV-negative disease^35^. This raises the question as to whether vaccines targeting tumour antigens can enhance T cell response and improve outcomes.

The range of antigens deliverable by vaccines is broad^36^ and can be categorized into those subject to thymic tolerance and those that are not. The former includes antigens such as mucin1, mesothelin, and carcinoembryonic antigen, whereas the latter includes T cell targets resulting from the mutational damage that cells undergo in the process of transformation, and virus-derived antigens from HPV. Although shared antigens can elicit immune responses^37–40^, T cells recognizing these antigens are often less effective at targeting tumour cells. This is largely due to a central T cell tolerance, a process during T cell development that eliminates high-affinity T cells specific for these ‘self-like’ antigens. As a result, the T cell repertoire is biased towards lower-affinity clones, limiting their capacity for effective tumour clearance and vaccine-induced expansion.

As in other cancers, efforts in HNSCC cancer have focused on targeting antigens arising from tumour-specific genomic alterations. If these genetic changes are transcribed and translated, the resulting proteins may be processed and presented on MHC molecules as peptides, termed neoepitopes. When recognized by T cells, the term ‘neoantigen’ is used, reflecting their immunogenicity. Although 0.1–1% of neoepitopes are immunogenic in patients, preclinical evidence has demonstrated that neoepitopes are immunogenic when delivered using a modified vaccinia Ankara (MVA) backbone^41^.

The TG4050 vaccine, sponsored by Transgene SA, based in an MVA platform, delivers up to 30 personalized neoantigens. In a phase I trial (NCT04183166), which began in 2019, patients with newly diagnosed operable, high-risk, HPV-negative stage III/IV HNSCC were enrolled and tissue was collected at surgeries for vaccine production. Patients who remained disease-free after adjuvant chemoradiotherapy, and for whom vaccine production was successful, were randomized to vaccination or observation. For the latter, the vaccine was stored for administration upon disease recurrence alongside standard systemic anticancer treatment. Among 16 patients per arm, disease-free survival (DFS) at 2 years was 100% in the vaccinated group compared with 81% in the controls^42^. The vaccine was well tolerated and vaccine-induced cytotoxic T cell responses were observed with persistence beyond 1 year following the last dose. A phase II trial has completed recruitment and clinical readouts are expected in 2027, and a randomized phase III trial is in the planning stage. Targeting viral antigens offers an ‘off-the-shelf’ alternative, with faster and less resource-intensive delivery. A phase I/II trial (NCT03418480) tested an mRNA vaccine, developed by BioNTech (BNT113), targeting HPV16 E6/E7, on 20 of 30 patients (20 with OPSCC). The vaccine was administered intravenously on eight occasions, with optimal maintenance dosing for up to 1 year in patients demonstrating favourable clinical outcomes. At the end of treatment, the disease control rate was 71%^42^. Using the interferon (IFN)g Elispot assessment, all evaluable patients had detectable HPV E6 and/or E7 vaccine responses, in either CD4^+^ or CD8^+^ or both sets of circulating T cells; these T cells expressed the activation markers programmed cell death protein 1 (PD-1), CD137, and CD57. TCR clonotype analysis demonstrated the expansion of both pre-existing and *ex novo* T cell responses in the blood. Paired biopsies from four patients demonstrated postvaccination expansion of T cell clonotypes within tumour tissue^43^.

BNT113 is being investigated further in the Ahead-Merit phase II registrational trial (NCT04534205), testing aPD-1 +/− vaccine patients with recurrent/metastatic PD-L1 + HPV16 + HNSCC. Separately, the phase II Versatile-002 trial (NCT04260126) from PDS Biotech reported a median OS of 39 months with an HPV16 peptide vaccine + aPD-1, compared with 17.9 months with aPD-1 alone in historical controls, in 53 patients with HPV16 + disease with a combined positive score (CPS) of > 1%. These results strongly suggest that aPD-1 combined with an HPV16 vaccine may offer clinical benefit in this patient group.

An emerging strategy is to target antigens resulting from tumour-associated metabolic stress rather than genetic mutation. The Modi-1 vaccine (Scancell, UK), under investigation in the Modify trial (NCT05329532), targets citrullinated peptides from vimentin and alpha-enolase, non-genomically encoded antigens presented via MHC class II. The vaccine is designed to harness the natural immune response to stressed cells, primarily mediated by cytotoxic CD4 T cells and direct this response to tumour cells. Following vaccination, modified peptides are presented on the cell surface via MHC class II antigens, activating tumour-specific CD4 T cells. Antigen recognition at the tumour site leads to release of proinflammatory cytokines, which promotes MHC class II upregulation on tumour cells. This establishes a positive feed-forward loop, enabling the presentation of endogenous tumour-derived peptides and facilitating direct cytotoxic activity by activated CD4 T cells^44^. Early clinical data show objective responses when the vaccine is combined with aPD-1 (personal communication, Scancell). A phase II trial in the neoadjuvant setting comparing aPD-1 with aPD-1 + Modi-1 is in preparation to explore further the mode of action and clinical effects in patients with HPV-negative HNSCC.

### Colorectal cancer

The BNT-122 (autogene cevumeran) trial represents one of the most exciting developments in the adjuvant treatment of colorectal cancer in recent years. Developed by BioNTech in collaboration with Genentech/Roche, this innovative approach uses personalized mRNA vaccine technology to induce antitumour immune responses against individual-specific tumour neoantigens. For surgeons managing high-risk colorectal cancer, the promise of a personalized immunotherapeutic strategy offers a potential paradigm shift in postoperative care. At the heart of this strategy is the use of ctDNA to identify patients at high risk of recurrence following surgery and chemotherapy. Patients who remain ctDNA-positive after completing standard treatment are thought to harbour micrometastatic disease, placing them at significant risk of relapse. BNT-122 is designed specifically for this group. After identifying residual tumour-specific neoantigens through genomic analysis of the resected tumour, a personalized mRNA vaccine encoding up to 20 neoantigens is manufactured for each patient. The vaccine is then delivered intravenously across 15 doses, beginning roughly 7 weeks after adjuvant chemotherapy, with the aim of generating a potent, tumour-specific T cell response to clear microscopic disease.

The BNT122-01 trial is a phase II, randomized, open-label, multicentre study comparing this personalized vaccine with standard surveillance in ctDNA-positive patients with stage II/III colorectal cancer and has now completed recruitment. The primary endpoint is DFS, with secondary endpoints including OS and ctDNA dynamics. Recruitment targets were ambitious: of an estimated 2750 screened patients, around 164 were to be randomized. The study was conducted across approximately 90 international sites, including 13 NHS centres, and forms part of the UK Cancer Vaccine Launchpad—a strategic initiative to streamline delivery of personalized vaccine trials across the NHS.

For surgeons, BNT-122 represents a tangible example of how immunogenomics is reshaping adjuvant therapy. Beyond curative resection and multidisciplinary team decision-making, surgeons now have a critical role in identifying eligible patients, supporting ctDNA screening, and integrating vaccine trials into the postoperative pathway. If the trial demonstrates a meaningful improvement in DFS, BNT-122 could herald a new standard of care in the management of minimal residual disease in colorectal cancer—with implications as profound as those seen in breast cancer or melanoma immunotherapy. Results from BNT122-01 are expected in 2025–2026. Until then, its ongoing delivery marks an important step in embedding precision immunotherapy into routine surgical oncology practice.

### Bladder cancer

#### Peptide-based vaccines

Peptide-based vaccines represent one approach to immunotherapy for bladder cancer, aiming to stimulate tumour-specific T cell responses. These vaccines utilize either tumour-associated antigens (self-antigens overexpressed in tumour tissue) or tumour-specific neoantigens (exclusively expressed by tumour cells).

One such study used personalized peptide vaccination (PPV), which involves the subcutaneous administration of immunogenic peptides chosen as potential antigens. In a phase I study of PPV, 10 patients with chemotherapy-refractory disease were treated with 12-weekly vaccinations using T cell-reactive peptides comprising 14 and 16 peptides restricted to HLA A24 and A2, respectively. PPV was well tolerated with no major adverse effects. Among 10 patients, 1 achieved a complete response (CR), 1 a partial response (PR), 2 had stable disease, and 6 showed progressive disease. Notably, durable responses were observed up to 24 months. Enhanced CTL responses and raised antipeptide IgG titres were observed in eight patients after treatment^45^.

In a subsequent phase II study involving 25 therapy-resistant patients with urothelial cancer (UC), PPV demonstrated a median OS of 11.3 months^46^. Another phase II trial compared PPV plus best supportive care (BSC) *versus* BSC alone in 80 patients with progressive metastatic UC (mUC) after platinum therapy. Although PPV did not significantly improve progression-free survival (PFS: HR 0.7, 95% confidence interval (c.i.) 0.4 to 1.2; *P* = 0.17), it significantly prolonged OS (7.9 *versus* 4.1 months; HR 0.58, 95% c.i. 0.34 to 0.99; *P* = 0.049)^47^.

Finally, PPV has also been evaluated in patients with metastatic upper tract UC in a single-arm phase II study. Patients received four immunogenic peptides over six subcutaneous doses. Median OS was 4.5 months with PPV alone and 13 months when combined with salvage chemotherapy. Peptide-specific CTL responses were observed in 46% of patients and correlated with longer survival (HR 0.37, 95% c.i. 0.16 to 0.85; *P* = 0.019)^45^.

#### Nucleic acid-based vaccines and combination strategies

To overcome tumour heterogeneity, immune escape, and T cell anergy, cancer vaccines comprising peptides or nucleic acid-based mRNA vaccines are being combined with immune checkpoint inhibitors (ICIs) with the goal of enhancing tumour-specific T cell activity.

NEO-PV-01, a personalized neoantigen peptide vaccine, was evaluated with nivolumab in a phase Ib trial in patients with metastatic melanoma, non-small cell lung cancer (NSCLC), and mUC (15 with mUC). A vaccine containing up to 20 unique peptides was administered subcutaneously into separate sites as five priming and two booster doses over 3 months. Nivolumab was given before, during, and after vaccination. In patients with mUC, the combination had an overall response rate (ORR) of 27%, median PFS of 5.8 months, median OS of 20.7 months, and a 1-year OS rate of 67%. Neoantigen-specific cytotoxic T cells were detected in peripheral blood^48^. Future efforts are focused on developing this vaccine therapy for melanoma and lung cancer as results in patients with mUC were similar to those achieved by ICI alone. However, outcomes were comparable to ICI monotherapy, limiting further development in mUC.

PGV009, a peptide vaccination, has also been studied in combination with the anti-PD-L1 antibody atezolizumab with PGV009, a peptide vaccine comprising 10 unique peptides^49^. Out of 12 enrolled participants, personalized vaccines were successfully manufactured for 10, with a median production time of approximately 20 weeks. The treatment was generally well tolerated; the most common adverse events were mild (grade 1) injection site reactions, fatigue, and fever. One patient experienced grade 3 immune-related hepatitis. All participants demonstrated the emergence of neoantigen-specific T cell responses during treatment, indicating that the vaccine effectively stimulated an immune response against tumour-specific antigens. In the adjuvant setting, among four patients treated after surgery, three remained free of disease recurrence at a median follow-up of 39 months. Among five patients with mUC who received atezolizumab + vaccine, two achieved radiological responses.

TAS0313, a multiepitope long-peptide vaccine, was tested with pembrolizumab in ICI-naïve patients with UC who had progressed on platinum-based chemotherapy. In this phase I/II study, TAS0313 was administered subcutaneously in priming and maintenance doses alongside pembrolizumab every 3 weeks. Among 30 patients, the ORR was 33% (17% CR, 17% PR), with a disease control rate of 67%. Median PFS was 5.0 months; OS data were immature, but the 1-year OS rate was 74%. No grade ≥ 3 adverse events were reported, supporting the safety of this regimen^50^. Additional studies evaluating this combination in ICI-naïve patients with UC are expected.

Given the challenges of rescuing immune responses with neoantigen vaccines in the setting of metastatic disease, efforts have shifted to the post-cystectomy space. A phase II trial (NCT06534983) is now evaluating autogene cevumeran plus nivolumab *versus* nivolumab alone as adjuvant therapy for high-risk muscle-invasive UC^51^. Eligible patients must be ICI-naïve with (y)pT3-4 or (y)pN + M0 disease after resection. The primary endpoint is DFS; secondary endpoints include OS, PD-L1-stratified DFS, and immune correlates^52^.

A second neoantigen vaccine, mRNA-4157, a lipid-encapsulated personalized vaccine encoding up to 34 neoantigens, was evaluated in the phase I KEYNOTE-603 trial. It was administered as adjuvant monotherapy (16) or in combination with pembrolizumab (63), including six patients with mUC. In the combination cohort, pembrolizumab was initiated two cycles before the introduction of mRNA-4157, which was given intramuscularly every 3 weeks for up to nine cycles^53^. At 8 months median follow-up, 92% of patients in the adjuvant cohort remained recurrence-free. Among the mUC group, three achieved CR and two PR. The combination was well tolerated, with no grade ≥ 3 adverse events. Neoantigen-specific T cell responses were confirmed via IFN-γ ELISpot^54^. These promising results are being evaluated in a randomized phase I/II study of adjuvant mRNA-4157 in combination with pembrolizumab and in the perioperative setting combining mRNA-4157 with enfortumab vedotin and pembrolizumab (InterPath-005, NCT06305767)^55^. Safety and activity data with these mRNA vaccines for patients with high-risk UC are awaited.

### Melanoma

Melanoma, a highly immunogenic skin cancer, has long been a prime candidate for vaccine-based immunotherapy. Although checkpoint inhibitors such as pembrolizumab and nivolumab have transformed treatment, up to 50% of patients fail to respond or experience relapse. Two principal approaches are currently under investigation for melanoma.

V940 (mRNA-4157) is an individualized mRNA vaccine developed by Moderna and Merck, encoding from 8 to 34 tumour-specific neoantigens. In the KEYNOTE-942 Phase 2b trial, 157 patients with resected stage III/IV melanoma received V940 plus pembrolizumab or pembrolizumab alone. The combination reduced the risk of recurrence or death by 44% (HR 0.56; *P* = 0.027). RFS at 18 months was 79% *versus* 62%^46^. Updated 3-year data showed a 49% reduction in recurrence or death, with 75% of patients recurrence-free at 2.5 years *versus* 56% with pembrolizumab alone. Grade 3 or higher adverse events occurred in 25% of the combination group compared with 18% receiving monotherapy^56^. A global phase 3 study (V940-001/INTerpath-001) has now completed recruitment to assess efficacy in stage IIB–IV melanoma^57^. The first interim data analysis is awaited.

SCIB1 is a DNA plasmid vaccine developed by Scancell using the ImmunoBody® platform, targeting gp100 and TRP-2 antigens found on melanoma tumour cells. SCIB1 is HLA restricted. iSCIB1+ has been developed, which relaxes the HLA restriction to allow unselected participation in the current clinical trial and is being evaluated in this context. In initial results in combination with checkpoint inhibitors (ipilimumab and nivolumab), SCIB1 demonstrated an 82% OR rate among 13 evaluable patients with unresectable disease, exceeding predefined efficacy targets^58^. Updated results presented after 25 patients had reached 25 weeks in the study showed a 72% ORR, with 20% of patients demonstrating a CR. A vaccine-specific T cell response was demonstrated in 94% of clinical responders. The vaccine did not appear to exacerbate immune-mediated adverse events when added to ipilimumab and nivolumab^59^. The vaccine currently continues under investigation in the Phase 2 SCOPE trial, supported by the UK’s Cancer Vaccine Launch Pad^60^.

BNT111 (FixVac) from BioNTech is an mRNA vaccine encoding four shared melanoma antigens (NY-ESO-1, MAGE-A3, tyrosinase, and transmembrane phosphatase with tensin homology). In the Lipo-MERIT Phase 1 study, a safety and efficacy assessment of the mRNA-based cancer vaccine of 89 patients with advanced melanoma was conducted. The vaccine was well tolerated, with no dose-limiting toxicities observed. Efficacy was assessed in 42 checkpoint inhibitor-experienced patients. Among 25 patients receiving BNT111 monotherapy, 3 had PRs, 7 had stable disease, and 1 achieved complete metabolic remission. In 17 patients receiving BNT111 plus anti-PD-1, 6 showed PRs. Treatment led to expansion and activation of tumour-antigen-specific memory T cells with a Th1 phenotype and strong cytotoxic activity. Notably, vaccine-induced memory T cells persisted for over 1 year with ongoing monthly vaccination^51^.

A randomized phase 2 trial has been completed in patients with melanoma who were refractory to or progressed after PD-1 therapy. This combined BNT111 with cemiplimab *versus* BNT111 alone or cemiplimab alone and enrolled 180 patients. This was announced as having met its endpoint with a statistically significant improvement in the ORR in the combination arm: 18% *versus* 17% *versus* 14%; *P* = 0.012^61^. However, in recently presented data, the median PFS in the three arms was remarkably similar (3.1, 2.8, and 3.2 months, respectively), calling into question the value of developing this approach further^62^.

## Cancer vaccines as part of combination therapy

As described earlier, vaccines often require co-administration with other agents to maximize their efficacy. A number of examples of this multiagent approach are given below.

### mRNA-4157/V940

The personalized mRNA-based neoantigen vaccine mRNA-4157/V940 targets patient-specific somatic neoantigens derived from cancer-specific mutations. These neoantigen peptide epitopes are absent from normal tissues and are therefore recognized as non-self by the host immune system, enabling highly tumour-selective immune responses with minimal risk of autoimmune toxicity. In this context, mRNA-4157/V940 has demonstrated encouraging activity when combined with immune checkpoint inhibition.

In resected stage III/IV melanoma, a global phase III trial is currently underway to assess its efficacy alongside pembrolizumab^63^. This follows earlier phase IIb data, which showed a 44% reduction in recurrence or death with the addition of the vaccine to pembrolizumab, compared with pembrolizumab alone^64^ highlighting the critical importance of multimodal immune modulation. Immune-mediated adverse event frequency was similar for the combination (37 (36%)) and monotherapy (18 (36%)) groups.

Several phase III trials using the same agent are also underway in NSCLC. One study investigates the use of mRNA-4157/V940, again targeting unique antigens within a patient’s own tumour, with pembrolizumab in the adjuvant setting after surgical resection^65^, whilst another includes patients with incomplete pathological responses after neoadjuvant aPD-1 therapy^66^.

A phase II/III trial in renal cell cancer is evaluating the same vaccine after nephrectomy in combination with pembrolizumab^67^, supported by data showing robust T cell responses from neoantigen peptide vaccines in kidney cancer^68^.

The adjuvant setting is a preferred window for intervention, as it allows for high-quality surgical tissue procurement, targeting minimal residual disease, and avoids the immunosuppressive effects often seen in the advanced setting.

### Autogene cevumeran (BNT122)

Autogene cevumeran (BNT122) is another individualized mRNA-based vaccine encoding personalized tumour neoantigens selected from a patient’s resected tumour.

**Fig. 3:**
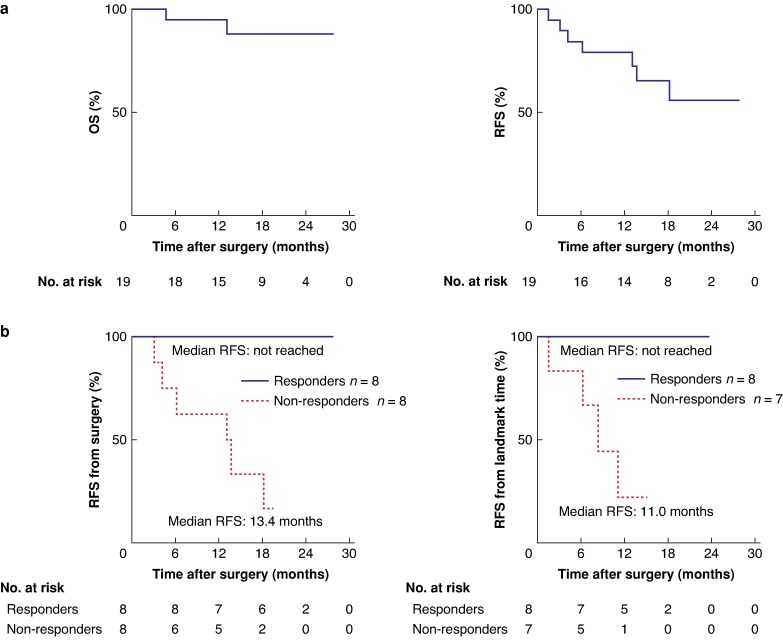
Kaplan–Meier plots taken from Rojas *et al.*^26^ demonstrating mRNA vaccine response and its correlation with delayed pancreatic cancer recurrence after surgery **a** OS; RFS. Median follow-up 19 months (*n* = 19). **b** RFS from surgery: median follow-up 18 months; HR 0.08 (95% c.i. 0.01 to 0.4); *P* = 0.003. RFS from landmark time: median follow-up 18 months; HR 0.06 (95% c.i. 0.008 to 0.4); *P* = 0.008. mRNA, messenger RNA; OS, overall survival; RFS, relapse-free survival; HR, hazard ratio; c.i., confidence interval.

In pancreatic cancer, a phase II trial is testing the combination of autogene cevumeran, atezolizumab, and chemotherapy in the adjuvant setting following surgical resection^68^. This follows previous data demonstrating robust immune responses and RFS benefit in patients who generated a substantial neoantigen-specific T cell response to the vaccine: at 18-month median follow-up, responders had a longer median RFS (not reached) compared with non-responders (13.4 months; *P* = 0.003)—*Fig. 3*^26^.

In bladder cancer, autogene cevumeran is being investigated in a phase II trial combined with nivolumab after cystectomy in patients with high-risk muscle-invasive disease^52^.

In colorectal cancer, this vaccine is undergoing a phase I/II trial in the adjuvant setting following surgical resection, in combination with atezolizumab^69^. Previous research suggests early intervention and low tumour burden may enhance vaccine efficacy^70^.

### TAA therapies

TAA-based vaccines are ‘off-the-shelf’ products that target shared antigens commonly overexpressed in tumour cells. Their appeal lies in simplified manufacturing and broader applicability, bypassing the logistical delays of personalized vaccine development. However, because these antigens are often derived from non-mutated self-proteins, there is a higher risk of immune tolerance and lower tumour specificity, which may limit the treatment response.

IO102-IO103, a dual-target off-the-shelf vaccine directed against indoleamine 2,3-dioxygenase 1 and PD-L1, was tested in a global phase III study in combination with pembrolizumab in patients with advanced melanoma^71^. This followed encouraging results from a phase I/II trial, which showed an 80% disease control rate and significant T cell infiltration in nearly half of patients. The phase III trial demonstrated a trend in favour of adding the vaccine (median PFS 19.4 *versus* 11 months), although this narrowly missed statistical significance. The results of this were presented at ESMO 2025 by Hassel *et al.*^72^. Adagloxad simolenin (OBI-822) targets Globo-H, a glycosphingolipid overexpressed in several tumours. It is currently under phase III evaluation alongside standard adjuvant chemotherapy in triple-negative breast cancer.

## Cancer vaccines in a preventative setting

Individuals with Lynch syndrome (LS) harbour germline mutations in the DNA mismatch repair (MMR) genes, which impairs the ability to correct DNA replication errors. These accumulating and uncorrected errors predispose carriers to an increased risk of early-onset malignancy, including but not limited to colorectal cancer.

The LynchVax project aims to develop a prophylactic vaccine that primes the immune system to target precancerous cells presenting neoantigens—aberrant peptides arising from replication errors due to MMR deficiency. To identify targetable neoantigens, researchers collected precancerous colorectal polyps and matched normal adjacent tissue from LS carriers. An integrated multiomics approach was employed combining whole-genome sequencing (WGS), bulk RNA sequencing, and immunopeptidomics to identify systematically neoantigens derived from recurrent and/or shared mutations in patients with LS.

WGS and RNA sequencing facilitate comprehensive *in silico* neoantigen prediction at both genetic and transcriptomic levels, whereas immunopeptidomics remains the standard for experimentally confirming peptide presentation via the MHC class I pathway. Top-ranked neoantigens with high predicted binding affinity will advance to preclinical immunogenicity testing, where their capacity to elicit CD8^+^ T cell responses will be evaluated.

Challenges remain, such as the extreme polymorphism of HLA haplotypes, which complicates broad vaccine coverage in the population. However, advances in bioinformatics tools, high-throughput sequencing, and population HLA frequency databases may enable the design of an ‘off-the-shelf’ vaccine for broader patient benefit and accessibility.

## Future directions and current challenges

Over the last few years, significant advances have been made in the development of cancer vaccines. Preventative cancer vaccines have shown remarkable progress and the potential to reduce global cancer incidence. Virus-like particle-based vaccines, such as those for HPV, HCV, and HBV, have effectively prevented infection and significantly reduced the risk of associated cancers. Vaccines for LS take this concept further, by priming immune surveillance for future cancer cells developing in the background of non-infectious tissues.

One major challenge for cancer vaccines is ensuring these vaccines induce long-lasting immune memory. This raises issues around booster vaccinations, which introduce logistical and financial burdens. Another major issue is identifying universal TAAs and stratifying high-risk populations, including those with genetic predispositions or early lesions. Understanding the immune microenvironment in premalignant stages will be essential.

Therapeutic cancer vaccines offer cautious promise, particularly in the adjuvant setting. Personalized vaccines are more specific and adaptable but face cost, infrastructure, and scalability challenges. Streamlining manufacturing and leveraging personalized medicine advances will be vital for accessibility. Current regulatory frameworks also struggle to classify and appropriately manage these contemporary technologies. For example, personalized mRNA cancer vaccines are regulated in the UK as advanced therapy medicinal products, subclassified as gene therapies. This places them in the same regulatory and governance category as chimeric antigen receptor T cell therapy, despite their wildly differing safety and tolerability profiles. Because they do not integrate into the genome, this classification is being reconsidered but the current classification presents an additional burden to widespread rollout. A proposed subcategory for non-genome-integrating nucleic acid therapies could facilitate a more efficient, risk-based approval pathway.

Shared neoantigen vaccines offer potential for ‘off-the-shelf’ mass production and broader applicability, but tumour heterogeneity and patient-specific MHC variation may impact efficacy. MHC variability also affects the risk of off-target immune responses.

Monitoring acquired immunity will be essential to assess vaccine efficacy and improve future iterations. Biomarker immunoprofiling, robust immune response assays, and deeper insight into individual responses are still developing. The US National Institute of Health has initiated a collaborative biomarker network, and international partnerships, including UK participation, could significantly accelerate cancer vaccine development.

Production is also highly dependent on strong multidisciplinary team working. Fresh-frozen tissue is strongly preferred over formalin-fixed samples, as it preserves DNA and RNA integrity. RNA degradation, in particular, can compromise expression analysis, which is needed for filtering viable candidates. Similarly, if the tissue is small or poorly preserved, it may not yield enough material for sequencing and prediction. Surgeons therefore have a crucial role in anticipating the potential for immunogenomic analysis during the procedure: ensuring adequate sample size, coordinating with pathology and molecular teams, and arranging prompt transport to preserve nucleic acids. In centres offering vaccine trials, preoperative planning should include clear tissue handling protocols.

## Conclusion

Cancer vaccines represent a promising new strand of cancer therapeutics, with the potential to induce durable tumour-specific immune responses. Although current applications are largely investigational, ongoing progress in antigen selection, delivery platforms, and clinical integration suggests they may play a role in future solid tumour management. Surgeons should remain engaged with developments in this field, as cancer vaccines may be incorporated into future treatment pathways and patient outcomes.

## Supplementary Material

zrag041_Supplementary_Data

## Data Availability

No data repository has been used for this manuscript.
