# Supplementary material for: Cancer vaccines: comprehensive review
Source: BJS Open. 2026 May 29;10(3):zrag041. doi: 10.1093/bjsopen/zrag041 (PMC13220758; doi:10.1093/bjsopen/zrag041)
Supplement: zrag041_Supplementary_Data [file zrag041_supplementary_data.docx]

**Glossary**

***Tumour-associated antigens (TAAs)*** - A protein or other molecule that is found on the surface of a tumour cell and can be recognised by the immune system and distinguished from healthy cells.

***Antigen-presenting cells (APCs)*** - Immune cells that present foreign antigens to T cells to initiate an adaptive immune response.

***Neoantigens*** - A novel antigen created by a tumour-specific mutation which is highly specific to a tumour cell

***Major histocompatibility complex (MHC) 1*** - Major Histocompatibility Complex Class I molecules are proteins found on the surface of nearly all nucleated cells. They present fragments of proteins (antigens) from inside the cell to cytotoxic T lymphocytes (CD8+ T cells). In cancer, MHC I presentation of tumor antigens is critical for the immune system to recognize and destroy malignant cells.

***Major histocompatibility complex (MHC) 2*** - Major Histocompatibility Complex Class II molecules are proteins expressed mainly on specialized immune cells such as dendritic cells, macrophages, and B cells. They present antigens from outside the cell to helper T lymphocytes (CD4+ T cells). In cancer vaccines, MHC II presentation helps activate and coordinate immune responses, including antibody production and cytotoxic T cell activation.

***Peptide / protein based vaccine*** - Vaccines produced by using of short fragments of proteins or whole proteins from tumour antigens to stimulate an immune response.

***DNA/RNA based vaccine*** - Vaccines produced by using of genetic material encoding tumour antigens to train immune cells.

***Cell based vaccine*** - Vaccines produced by using whole cells, often dendritic cells, to present tumour antigens.
